# Draft genome sequence of a multidrug-resistant *Streptococcus gallolyticus* strain MTR-Sg-DF01 isolated from an apparently healthy domestic duck

**DOI:** 10.1128/mra.00720-26

**Published:** 2026-09-23

**Authors:** Dilruba Akter Jany, Md. Abdullah Evna Hasan, Zuhayr Bakhtiyar, Md. Tabeer Hossain Antor, Sadia Afrin Punom, Atikur Rahaman, Md. Monirul Islam, Anindita Ash Prome, Sukumar Saha, Md. Tanvir Rahman

**Affiliations:** 1 Department of Microbiology and Hygiene, Faculty of Veterinary Science, Bangladesh Agricultural University 54492, Mymensingh, Bangladesh; Portland State University, Portland, Oregon, USA

**Keywords:** whole-genome sequencing, *Streptococcus gallolyticus*, multidrug resistance, domestic duck, antimicrobial resistance, Bangladesh

## Abstract

We report the draft genome sequence of a multidrug-resistant *Streptococcus gallolyticus* strain MTR-Sg-DF01, isolated from the feces of an apparently healthy duck. The 2.05-Mb genome harbored the resistance genes *qac*G, *van*T, and *van*Y, but lacked plasmids, and encoded a Bovicin 255 biosynthetic cluster, highlighting its relevance to zoonotic AMR surveillance.

## ANNOUNCEMENT

*Streptococcus gallolyticus,* a member of the *Streptococcus bovis/Streptococcus equinus* complex, is an opportunistic pathogen associated with bacteremia, infective endocarditis, and colorectal cancer in humans (1–3). Here, we report the draft genome sequence of a multidrug-resistant *S. gallolyticus* isolated from an apparently healthy domestic duck (*Anas platyrhynchos domesticus*) in Mymensingh, Bangladesh.

On 7 June 2025, a freshly voided fecal sample was collected from an apparently healthy domestic duck at Ganginarpar, Mymensingh, Bangladesh (24.7572°N, 90.4067°E) using a sterile cotton bud and placed into a test tube containing 5 mL of nutrient broth (HiMedia, India, catalog no. M002), and then incubated aerobically at 37°C overnight. Therefore, the culture was streaked onto *Enterococcus* agar (HiMedia, India) and repeatedly subcultured until a pure colony was obtained. The isolate produced smooth, grayish-white colonies on *Enterococcus* agar and gram-positive cocci on Gram staining (4). Species identity was confirmed by whole-genome sequencing. Antimicrobial susceptibility was determined by the Kirby–Bauer disk diffusion method according to CLSI 2024 guidelines, which identified the isolate as multidrug-resistant (MDR), showing resistance to ampicillin, linezolid, and fosfomycin (5).

The isolate was then cultured in Luria–Bertani broth (BD, USA; catalog no. 244620). Genomic DNA was extracted using the DNeasy Blood & Tissue Kit (Qiagen, Germany; catalog no. 69504) according to the manufacturer’s instructions. Whole-genome sequencing on an Illumina NextSeq 2000 generated 730,239 paired-end (2 × 150 bp) read pairs (371.3 Mb). Adapter trimming and quality control were performed using fastp v0.23.4 (6), and *de novo* assembly was performed using SPAdes v3.15.5 (7). Genome annotation was performed using the NCBI PGAP v6.10 (8). Taxonomic classification and contamination screening were performed using Kraken2 v2.1.3 (9), whereas genome quality assessment and sequence typing were performed using CheckM v1.2.5 (10) and MLST v2.33.1 (https://github.com/tseemann/mlst). AMR genes, virulence genes, plasmids, bacteriocins, prophages, and pathogenicity were assessed using CARD-RGI (11), VFDB via ABRicate (12), PlasmidFinder (13), BAGEL4 (14), PHASTEST (15), and PathogenFinder (https://cge.food.dtu.dk/services/PathogenFinder/), respectively. Unless otherwise noted, default parameters were used for all software.

The *S. gallolyticus* strain MTR-Sg-DF01 genome characteristics are given in Table 1. The draft genome comprised 2,049,756 bp in 152 contigs, with 37.56% GC content, an N50 of 23,899 bp, an N90 of 7,670 bp, a largest contig of 83,384 bp, no ambiguous bases, and 170.8× post-trimming genome coverage.

**TABLE 1:** Genomic characteristics of *S. gallolyticus* strain MTR-Sg-DF01 isolated from an apparently healthy domestic duck

Attribute	Value
BioProject accession	PRJNA1366106
BioSample accession	SAMN53307292
GenBank/WGS accession	JBSWZM000000000
Assembly accession	GCA_054054565.1
SRA accession	SRR36096626
Number of spots	730,239
Number of bases	371.3 Mb
SRA data volume	123 MB
Genome size	2,049,756 bp
GC content	37.56%
Number of contigs	152
Largest contig	83,384 bp
N50	23,899 bp
N90	7,670 bp
L50	26
L90	85
Ns per 100 kbp	0.00
Sequencing depth	170.8×
Genes, total	2,606
Coding genes	2,403
CDSs, total	2,479
Protein-coding CDSs	2,276
RNA genes	126
rRNAs	69
5S rRNA	3
16S rRNA	15
23S rRNA	51
tRNAs	56
ncRNAs	1
Pseudogenes	203
Pseudogenes (frameshifted)	71
Pseudogenes (incomplete)	156
Pseudogenes (internal stop codons)	96
Pseudogenes (multiple problems)	112
Genome completeness	100.00%
Contamination	2.58%
Strain heterogeneity	68.75%
ANI	97.4993%
Closest reference genome	*Streptococcus gallolyticus* strain ICDDRB-NRC-S1 (GCF_001477575.1_ASM147757v1)
Matched fragments	512/610
MLST allelic profile	aroE (~31), glgB (~22), nifS (~31), p20 (~27), tkt (13), trpD (~25), uvrA (~34)
Number of ARGs	3
ARGs detected	*qacG*, *vanT*, *vanY*
Pathogenicity score	0.768
Matched pathogenic families	6
Matched non-pathogenic families	1
Input proteome coverage	0.34%
Number of bacteriocin clusters	1
Predicted bacteriocin	Bovicin 255 peptide
Bacteriocin cluster length	5,492 bp
BAGEL4 score	42.2

PGAP annotated 2,606 genes, including 2,479 CDSs, 2,403 coding genes, 2,276 protein-coding CDSs, 126 RNA genes, and 203 pseudogenes. RNA genes comprised 69 rRNAs (3 5S, 15 16S, and 51 23S rRNAs), 56 tRNAs, and 1 ncRNA. Pseudogenes included 112 with multiple annotation issues, 71 that were frameshifted, 156 that were incomplete, and 96 with internal stop codons. CheckM showed 100% completeness, 2.58% contamination, and 68.75% strain heterogeneity. Kraken2 and average nucleotide identity (ANI) analysis confirmed the isolate as *S. gallolyticus*, showing 97.5% identity to *S. gallolyticus* strain ICDDRB-NRC-S1 (RefSeq assembly GCF_001477575.1). Multilocus Sequence Typing (MLST) suggested a potentially novel allelic profile: aroE (~31), glgB (~22), nifS (~31), p20 (~27), tkt (13), trpD (~25), and uvrA (~34). CARD-RGI detected *qac*G, *van*T, and *van*Y. No plasmids, virulence genes, or prophages were detected. PathogenFinder predicted human pathogenic potential (score 0.768; 6 pathogenic and 1 non-pathogenic families), and BAGEL4 identified a 5,492-bp Bovicin 255 peptide cluster.

## Data Availability

The whole-genome shotgun project has been deposited in GenBank under accession number JBSWZM000000000. The genome assembly is available under accession number GCA_054054565.1. Raw sequencing reads are available under SRA accession number SRR36096626. Associated metadata are available under BioSample accession SAMN53307292 and BioProject accession PRJNA1366106.
